# Efficacy and Safety of a Mixture of Microencapsulated Sodium Butyrate, Probiotics, and Short Chain Fructooligosaccharides in Patients with Irritable Bowel Syndrome—A Randomized, Double-Blind, Placebo-Controlled Study

**DOI:** 10.3390/jcm14010006

**Published:** 2024-12-24

**Authors:** Anita Gąsiorowska, Marek Romanowski, Ewa Walecka-Kapica, Aleksandra Kaczka, Cezary Chojnacki, Milena Padysz, Marta Siedlecka, Julia Banasik, Aleksandra Sobolewska-Włodarczyk, Maria Wiśniewska-Jarosińska, Joanna B. Bierła, Nize Otaru, Bożena Cukrowska, Robert E. Steinert

**Affiliations:** 1 Gastroenterology Department, Medical University of Lodz, Pomorska 251, 92-213 Łódź, Poland; anita.gasiorowska@umed.lodz.pl (A.G.); rmarek30@poczta.onet.pl (M.R.); ewawk@op.pl (E.W.-K.); akaczka@wp.pl (A.K.); cezary.chojnacki@umed.lodz.pl (C.C.); milenapadysz@gmail.com (M.P.); martasiedlecka@poczta.onet.pl (M.S.); julia.a.banasik@gmail.com (J.B.); aleksandra.sobolewska-wlodarczyk@umed.lodz.pl (A.S.-W.); majkawj@bmp.net.pl (M.W.-J.); 2 Department of Microbiology and Clinical Immunology, The Children’s Memorial Health Institute, Aleja Dzieci Polskich 20, 04-730 Warsaw, Poland; j.bierla@ipczd.pl; 3 Health, Nutrition & Care, DSM-Firmenich, 4303 Kaiseraugst, Switzerland; nize.otaru@dsm-firmenich.com (N.O.); robert.steinert@dsm-firmenich.com (R.E.S.); 4 Department of Pathomorphology, The Children’s Memorial Health Institute, Aleja Dzieci Polskich 20, 04-730 Warsaw, Poland; 5 Department of Surgery, Division of Visceral and Transplantation Surgery, University Hospital Zürich, 8091 Zürich, Switzerland

**Keywords:** irritable bowel syndrome, probiotics, prebiotics, synbiotics, microencapsulated sodium butyrate

## Abstract

**Objective:** Biotics are increasingly being used in the treatment of irritable bowel syndrome (IBS). This study aimed to assess the efficacy and safety of a mixture of microencapsulated sodium butyrate, probiotics (*Lactocaseibacillus rhamnosus* DSM 26357, *Lactobacillus acidophilus* DSM 32418, *Bifidobacterium longum* DSM 32946, *Bifidobacterium bifidum* DSM 32403, and *Bifidobacterium lactis* DSM 32269), and short-chain fructooligosaccharides (scFOSs) in IBS patients. **Methods:** This was a randomized, double-blind, placebo-controlled trial involving 120 adult participants with IBS. The primary outcome of the 12-week intervention was the improvement in IBS symptoms and quality of life (QOL), assessed with the use of IBS-Adequate Relief (IBS-AR), IBS-Global Improvement Scale (IBS-GIS), IBS-Symptom Severity Score (IBS-SSS), and IBS-QOL. Secondary outcomes were the number and type of stools (assessed via the Bristol Stool Form scale), patient-recorded symptoms, anthropometric parameters, and levels of selected inflammatory cytokines. **Results:** As early as at 4 weeks, there was a higher percentage of patients in the biotic group reporting adequate relief of symptoms (based on IBS-AR) than in the placebo group (64.7% vs. 42.0%, respectively, *p* = 0.023). At 12 weeks, fewer patients in the biotic group reported a ‘worsening of symptoms’ (based on IBS-GIS) than in the placebo group (5.9% vs. 16.0% respectively, *p* = 0.015). There were no significant differences between groups in IBS-QOL or IBS-SSS or any of the secondary outcome measures except the patient-recorded ‘urgency to defecate’ (*p* = 0.015) at week 12, which was significantly lower in the biotic group. The intervention was safe and well tolerated. **Conclusions:** A biotic mixture consisting of microencapsulated butyrate, probiotics, and small amounts of scFOSs is safe and effective in improving gastrointestinal symptoms in patients with IBS.

## 1. Introduction

Irritable bowel syndrome (IBS) is a common functional gastrointestinal disorder with a prevalence ranging from less than 5% to as high as 20%, depending on the region and diagnostic criteria used [1,2]. IBS is characterized by abdominal pain associated with changes in the number of bowel movements and consistency of stool, bloating, and abnormal bowel habits and is typically classified into three main subtypes: diarrhea-predominant (IBS-D), constipation-predominant (IBS-C), and mixed bowel habits (IBS-M) [3].

The mechanisms of IBS are not fully understood, but various factors such as disturbances in gut motility, visceral hypersensitivity, psychological stress, altered immune function of the gut with mucosal inflammation, impaired epithelial barrier function, and the dysregulation of the microbiota–gut–brain axis affect the development of the disease [4,5]. In addition, intestinal dysbiosis, defined as microbial imbalance and changes in biodiversity and functional activity of the gut microbiota, has been increasingly recognized as a potential trigger for IBS [6]. Indeed, there is clear evidence of notable changes of gut microbiota composition of individuals with IBS [7]. A meta-analysis of studies examining microbiota associated with IBS revealed significantly lower number of *Bifidobacterium*, *Lactobacillus*, and *Faecalibacterium prausnitzii*, one of the primary butyrate producers in the intestine, among IBS patients compared to healthy individuals [8]. Furthermore, a decline in butyrate-producing bacteria has been specifically noted in the IBS-D and IBS-M subtypes [9].

Butyrate, a short-chain fatty acid (SCFA), is vital for gut health, acting as an energy source for colonocytes and supporting the integrity of the intestinal epithelial barrier [10]. It also exhibits anti-inflammatory properties and modulates immune responses by suppressing inflammatory cytokines and myeloperoxidase activity and inhibiting proliferation of pathogens [11]. The reduction of butyrate in IBS patients suggests a potential therapeutic role for targeted supplementation, particularly in the form of microencapsulated butyrate, which can ensure delivery to the distal parts of the intestine. Indeed, a previous study involving IBS patients who received microencapsulated sodium butyrate showed significant reductions in IBS symptoms [12].

Another potential strategy to increase intestinal SCFA levels, including butyrate, and address gut dysbiosis involves the use of probiotics, prebiotics, or synbiotics (a combination of probiotics and prebiotics) [13]. Probiotics are live microorganisms, most commonly bacteria, that provide health benefits when consumed in sufficient quantities [14], while prebiotics, including fructooligosaccharides (FOS), are substrates for the growth of host bacteria conferring a health benefit [15]. The recently published meta-analyses of randomized placebo-controlled clinical trials have demonstrated the effectiveness of probiotics, especially multi-strain, and synbiotics, but not prebiotics in improving symptoms in IBS patients [16,17].

Recently, we conducted a randomized double-blind placebo-controlled study that showed significant clinical improvement in IBS-D patients using a multi-strain synbiotic preparation containing a probiotic mixture of two *Lactobacillus* strains (*L. rhamnosus* DSM 26357 (formerly called FloraActive 19070) and *L. acidophilus* DSM 32418) and three *Bifidobacterium* strains (*B. longum* DSM 32946, *B. bifidum* DSM 32403, and *B. lactis* DSM 32269) along with short-chain FOSs (scFOS) [18]. This synbiotic was not only effective in symptom relief but also well-tolerated, indicating its potential as a therapeutic option for IBS-D. However, there is currently no research evaluating the combined efficacy of colon-targeted microencapsulated butyrate and probiotics or synbiotics in the treatment of IBS.

Therefore, this study aimed to assess the efficacy and safety of combining microencapsulated sodium butyrate with the previously tested synbiotic preparation in adult IBS patients.

## 2. Materials and Methods

### 2.1. Design of the Study

This was a randomized, double-blind, placebo-controlled trial carried out at outpatient clinics between April 2021 and December 2023. The detailed scheme and the protocol of the study was published in 2021 [19]. The study was registered in the ClinicalTrials.gov with the following identifier number: NCT05013060.

The intervention group (n = 60) received a biotic product containing of a mixture of microencapsulated butyrate, probiotic *Bifidobacterium* and *Lactobacillus* strains, and small amounts of prebiotic scFOSs (Table 1). The control group (n = 60) received maltodextrin as a placebo.

**Figure 1:**
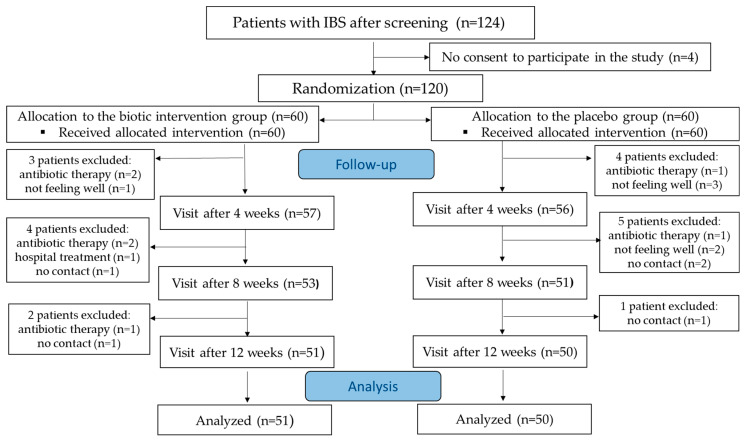
Study flowchart.

The duration of the intervention was twelve weeks. The study protocol included five visits: (i) the screening visit intended to qualify patients to be enrolled in the study, (ii) the baseline visit occurring up to two weeks after the screening visit, at which participants were randomized to study groups, and (iii) three follow-up visits (after a 1-week run-in period) at weeks 4, 8, and 12 ± 3 days after the start of the intervention (Figure 1). The visit at week 8 was a remote visit conducted by phone by a researcher and the remaining visits took place at the clinic.

Participants who were enrolled in the study were asked to fill out a diary on a daily basis containing information on the number of bowel movements, the consistency of stools, and the severity of the following gastrointestinal symptoms: pain, flatulence, and urgency to defecate. In addition, participants were monitored by interviewers via phone every week to document the intake of study product, daily diary entries and collection of data on adverse events (AEs), and the use of drugs, particularly antibiotics.

The study adhered to the ethical principles outlined in the Declaration of Helsinki and Good Clinical Practice guidelines. The protocol received approval from the Ethics Committee at the Medical University of Lodz, Poland (approval number RNN/213/20/KE).

All participants were fully informed about the study’s objectives, design, and procedures. Those who agreed to take part provided written informed consent before enrollment.

### 2.2. Randomization and Intervention

Participants (n = 120) were randomly allocated in a 1:1 ratio to the biotic or placebo group. The randomization into one of the two study groups was done by an independent statistician in blocks of four using a computer-generated randomization list. Allocation to study groups was blinded to both patients and investigators.

Subjects allocated to the biotic product group received capsules with a biotic formulation consisting of microencapsulated sodium butyrate, a multispecies probiotic including *Bifidobacterium (B.) lactis* DSM 32269, *B. longum* DSM 32946, *B. bifidum* DSM 32403, *Lactobacillus (L.) acidophilus* DSM 32418, and *Lacticaseibacillus (L.) rhamnosus* DSM 26357 (formerly called *L. rhamnosus* FloraActive 19070) as well as scFOS (Actilight^®^, Beghin Meiji, Marckolsheim, France). Microencapsulated sodium butyrate was formulated as a colon-release preparation using a pH-dependent polymer, anionic methacrylate copolymer (EmergoPharm Ltd., Konstancin-Jeziorna, Poland), which was validated for targeted colon delivery [20]. The exact composition of the biotic product is described in Table 1. Subjects allocated to the placebo group received capsules containing 440 mg of maltodextrin. All capsules were uniform in appearance and taste and were provided in identical packaging. The labels included the study title, Ethics Committee approval number, and the expiration date.

The biotic product or placebo were prepared, blinded, and supplied by DSM-Firmenich (Kaiseraugst, Switzerland). The formulation of the product was co-developed under a license with Nordic Biotic Ltd. (Warsaw, Poland).

### 2.3. Patients

The trial included adult participants diagnosed with IBS based on the Rome IV criteria, which require recurrent abdominal pain occurring at least once a week on average for the past three months, associated with two or more of the following criteria: related to defecation, associated with a change in the frequency of stool, or with a change in the form of stool [21]. A total of 189 IBS patients were screened, of which 124 met the study inclusion criteria. Of these, 120 were randomized and allocated to either the intervention group receiving the biotic mixture (n = 60) or to the control group receiving the placebo (n = 60). During the 12-month intervention, 19 patients (15.8%) did not complete the study, including nine from the biotic group (15.0%) and ten from the placebo group (16.7%). The study flowchart is presented in Figure 1.

#### 2.3.1. Inclusion and Exclusion Criteria

To be eligible for the trial, subjects had to meet all of the inclusion criteria and none of the exclusion criteria.

Inclusion criteria were as follows: (i) males and females aged from 18 to 70 years; (ii) IBS diagnosed based on the Rome IV criteria classified into three main subtypes: IBS-D, IBS-C, or IBS-M; (iii) IBS with at least moderate symptom severity, defined as an IBS-Severity Scoring System (IBS-SSS) score of >175 points [21]; (iv) good physical and mental condition, assessed based on the patient’s history and physical examination; (v) laboratory tests (complete blood count and blood chemistry panel) within normal limits or not to be clinically significant; (vi) provided written informed consent; and (vii) had the ability to adhere to the investigators’ instructions regarding study protocol and procedures.

Exclusion criteria included (i) unclassified IBS; (ii) gastrointestinal conditions other than IBS, such as clinical or endoscopic diagnosis of gastroenteritis, inflammatory bowel disease, or celiac disease; (iii) other diseases, such as cardiovascular disorders including uncontrolled hypertension (blood pressure > 170/100 mmHg), respiratory disorders (chronic obstructive pulmonary disease or asthma), endocrine disorders including diabetes mellitus (fasting blood glucose > 11 mmol/L) or thyroid diseases, severe neurological conditions, malignancy, and hepatic or renal impairment; (iv) pregnancy or breastfeeding; (v) lactose intolerance or hypersensitivity to soy or other food allergens; (vi) a surgical procedure scheduled during the clinical study; (vii) the use of following drugs: corticosteroids, antibiotics during one month preceding the study, gastrointestinal motility stimulants or dietary fiber supplements during the 2 weeks preceding the clinical study, and any medicines except contraceptive pills or intramuscular contraceptives, hormone replacement therapy, L-thyroxine, antidepressants at low doses (up to 25 mg of amitriptyline, nortriptyline, or selective serotonin reuptake inhibitor per day), antihypertensives at low doses (diuretics, angiotensin converting enzyme inhibitors, or angiotensin receptor antagonists), but they have been used at a stable dose and for at least 1 month prior to the study; (viii) the use of intestine microbiota-targeted dietary supplements or drugs (e.g., probiotics, prebiotics, synbiotics, or SCFAs) and declining to participate in a 1-month washout phase; (ix) being enrolled in another clinical trial within the past three months; (x) alcohol or substance abuse; and (xi) COVID-19 infection or contact with SARS-CoV-2-positive individuals during the previous 2 weeks.

#### 2.3.2. Withdrawal Criteria

Participants were removed from the study by the investigators for the following reasons: (i) withdrawal of informed consent; (ii) compliance with product/placebo supplementation below 80%; (iii) failure to attend study visits; (iv) inability to contact the telephone interviewer; (v) discovery of exclusion criteria after enrollment; (vi) occurrence of any serious AEs during the intervention period; and (vii) use of antibiotics during the study.

### 2.4. Compliance

Participants were given either the biotic product or placebo during each clinic visit and instructed to return the packaging and any unused capsules at their subsequent visit. Compliance was determined by the number of returned capsules, with a threshold of over 80% compliance required for continued participation in the study.

### 2.5. Measurements/Procedures

The following measurements/procedures were performed during the study as described in more detail previously [19]: (i) anthropometric measurements—at baseline and at weeks 4 and 12 of the intervention, including weight, height, body mass index (BMI), waist-to-hip ratio, and arm or calf circumference; (ii) disease symptom severity assessment—at baseline and at weeks 4, 8, and 12 of the intervention with the use of the IBS-SSS [22], and weekly with a patient-rated symptom severity Likert scale derived from participants’ diaries; (iii) assessment of improvement or worsening of IBS symptoms—at weeks 4, 8, and 12 of treatment with the use of the IBS-SSS, IBS-Global Improvement Scale (IBS-GIS) [23], and the IBS—Adequate Relief (IBS-AR) [24]; (iv) assessment of quality of life (QOF) at baseline and at weeks 4 and 12 with the use of the IBS-QOL questionnaire [25]; (v) assessment of the number of bowel movements and the consistency of stools reported in patients’ diaries; (vi) assessment of AEs using data from telephone interviewers, investigators, and patients’ diaries throughout the entire study; (vii) performing blood laboratory tests (complete blood count; liver enzymes (ALT, AST); bilirubin, amylase, creatinine, C-reactive protein, glucose, and electrolyte levels) at the screening visit; and (viii) measurement of serum cytokines interleukin 6 (IL-6) and chemokine (C-C motif) ligands 4 (CCL4) previously known as macrophage inflammatory protein (MIP-1β) at baseline and at weeks 4 and 12 of intervention.

#### 2.5.1. Questionnaires

IBS-AR is a binary question that asks participants if they experienced adequate relief from IBS symptoms over the past week (seven days) [24]. The response options are “YES” or “NO”.

The IBS-SSS is a 5-item survey assessing the severity of specific symptoms over the past ten days, including: abdominal pain (IBS-SSS1), frequency of abdominal pain, i.e., the number of days with abdominal pain (IBS-SSS2), severity of flatulence (IBS-SSS3), dissatisfaction with bowel habits (IBS-SSS4), and the impact on quality of life (IBS-SSS5) [22]. Each of the five questions can yield a maximum score of 100 points (calculated by multiplying the number of days with abdominal pain by 10). Total scores range from 0 to 500, with higher scores indicating more severe symptoms.

The IBS-GIS includes a question about how patients perceive changes in the severity of their global IBS symptoms over the past seven days compared to before the intervention. Responses are recorded using a 7-point Likert scale defined by the patient, with higher scores indicating greater improvement in global symptoms.

The IBS-QOL is a 34-item questionnaire that evaluates how much IBS affects a patient’s quality of life [25]. Each item is scored on a 5-point Likert scale, resulting in a total score ranging from 34 to 170. Higher scores indicate a worse quality of life.

Disease symptom severity, as recorded in patient diaries (except for the feeling of incomplete evacuation after a bowel movement), was assessed using a patient-reported symptoms scale with a 5-point Likert scale. A score of 0 indicated no symptoms, while scores from 1 to 4 reflected varying levels of symptom severity, with higher scores indicating worse symptoms. The sensation of incomplete evacuation after a bowel movement was evaluated as either YES or NO.

The number of bowel movements was recorded in patient diaries. Additionally, stool consistency was evaluated using the Bristol Stool Form (BSF) scale, which categorizes stools into seven types: types 1–2 indicate constipation, types 3–4 are considered “normal”, and types 5–7 suggest diarrhea [26].

#### 2.5.2. Cytokine Measurement

Cytokines were assessed in patients’ sera by an immunoenzymatic technique with the use of commercial kits (Human IL-6 ELISA and Human CCL-4 ELISA, Biorbyt, Cambridge, UK) according to the manufacturer’s instructions.

#### 2.5.3. Monitoring of Adverse Events

AEs were monitored throughout the study by investigators and telephone interviewers. Patients reported the occurrence of AEs in diaries. The results were presented as AE incidence and AE absolute prevalence [27]. AE incidence was calculated as a percentage of patients who reported AEs out of the total number of participants in the biotic or placebo group [28]. AE absolute prevalence was calculated as a percentage of the days during which patients reported AEs out of the total number of days spent during the intervention (12 weeks × 7 days = 84 days) [28].

### 2.6. Outcomes

#### 2.6.1. Primary Endpoints

The main efficacy endpoint of the intervention was the improvement in the severity of IBS symptoms, assessed with the use of questionnaires and scoring validated by international experts and recognized as suitable for such trials [16,17,22,23,24,29,30], i.e., IBS-AR [24], IBS-SSS [22], and IBS-GIS [23]. In addition, quality of life was assessed using the IBS-QOL questionnaire [25]. The use of three questionnaires to evaluate clinical improvement (or in the case of the IBS-GIS scale, also deterioration) was based on the protocol of our previous study that assessed the efficacy of a synbiotic preparation with a similar combination of probiotics and prebiotics, but without microencapsulated butyrate [18]. To examine whether adding microencapsulated butyrate impacts the intervention’s effectiveness, the same parameters were analyzed. The detailed protocol for the current study, including a thorough description of its endpoints, has been published [19] and registered on ClinicalTrials.gov (registry number NCT05013060).

The following criteria for an improvement of clinical symptoms were used: (i) IBS-AR—patients that for the last seven days had experienced “adequate relief” (who responded “YES”); (ii) IBS-SSS (both total score and sub-scoring for specific symptoms)—a drop of at least 50% of scoring points compared with baseline; and (iii) IBS-GIS—a score of >4 points for an improvement (and <4 points for a worsening). Of note, taking a 50% drop in the IBS-SSS score as the cut-off represents a restrictive approach, as some researchers consider a drop in the number of points by at least 30% compared to baseline [16,17,24] or a reduction in the number of points by 50, but only in the case of the total IBS-SSS score, not in the IBS-SSS1-SSS5 subscales [24].

#### 2.6.2. Secondary Endpoints

Secondary efficacy endpoints included the effect of the intervention on (i) disease symptom severity using patient-recorded symptoms scales; (ii) the number of bowel movements per day and the consistency of stools assessed using the BSF scale [26]; (iii) anthropometric measurements and BMI; and (iv) serum cytokine levels (IL-6 and CCL-4).

### 2.7. Statistics

#### 2.7.1. Sample Size Calculation

Sample size calculation was based on the anticipated difference in improvement (% of patients) of IBS symptom severity between groups. Assuming a difference between groups of 20% in addition to the well-known placebo effect in control groups of 30% (and with an assumed alpha error of 5%, statistical power of 80%, and data loss of 20%), a minimum sample size of 55 patients was calculated using Statistica software version 14.0 (Tibco Software Inc., Palo Alto, CA, USA). To increase statistical power, the sample size was set at 60 participants per group, with a total of 120 patients planned for inclusion in the study.

This approach was in line with calculations based on absolute values for symptom severity using IBS-SSS data from our previous study [18] that suggested a difference in change from baseline for total IBS-SSS scores of 27.6 points when compared with a placebo [18]. Assuming mean values of 170.0 and 142.0 at the end of intervention for the biotic and placebo group, respectively, and a common standard deviation (SD) of 95.4 points, a minimum sample size of 116 patients was calculated.

Of note, calculations based on absolute values for IBS-QOL data also suggested a similar number patients to be included. Our previous study suggested mean values of 55.0 and 44.0 at the end of intervention for the placebo and biotic group, respectively, and with a common SD of 18.0 and the same values for alpha error, statistical power, and drop out, a minimum sample size of 106 patients was calculated.

#### 2.7.2. Statistical Analyses

All statistical analyses were performed using Stata version 16.0 (StataCorp LLC, Lakeway, TX, USA). Fisher’s exact test or McNemar’s test was applied to nominal variables (e.g., adverse event occurrence and the presence or absence of symptoms). Unpaired/paired *t*-tests were used for continuous variables with normally distributed residuals (as tested by the Shapiro–Wilk test), while non-normally distributed data were analyzed with two-sample Wilcoxon signed-rank tests. Daily data from patient diaries were aggregated into weekly observations. The analysis followed the per-protocol approach, with a significance threshold set at 0.05 for all tests.

## 3. Results

### 3.1. Patients’ Characteristics

A total of 101 patients (84.2%) completed the study and were included in the per-protocol analysis (Figure 1). Patients’ characteristics are shown in Table 2. There were no significant differences between groups in terms of sex, age, and anthropometric measurements and BMI or IBS type and severity, with females being predominant in both groups, at 78.4% and 70.0% in the biotic and the placebo group, respectively.

### 3.2. Primary Outcomes: Improvement in Severity of IBS Symptoms and Quality of Life

The proportion of patients with an improvement in IBS symptoms measured as adequate relief using the IBS-AR scale was significantly higher in the biotic group compared with the placebo at week 4 (*p* = 0.023, with an odds ratio (OD) of 2.53 [95% of confidence interval (CI) 1.13–5.65] (Table 3). At week 8 and 12 of the intervention, the percentage of patients reporting relief increased to 74.5% and 72.5% in the biotic group and 62.0% and 56.0% in the placebo group, but these differences did not reach statistical significance.

**Figure 2:**
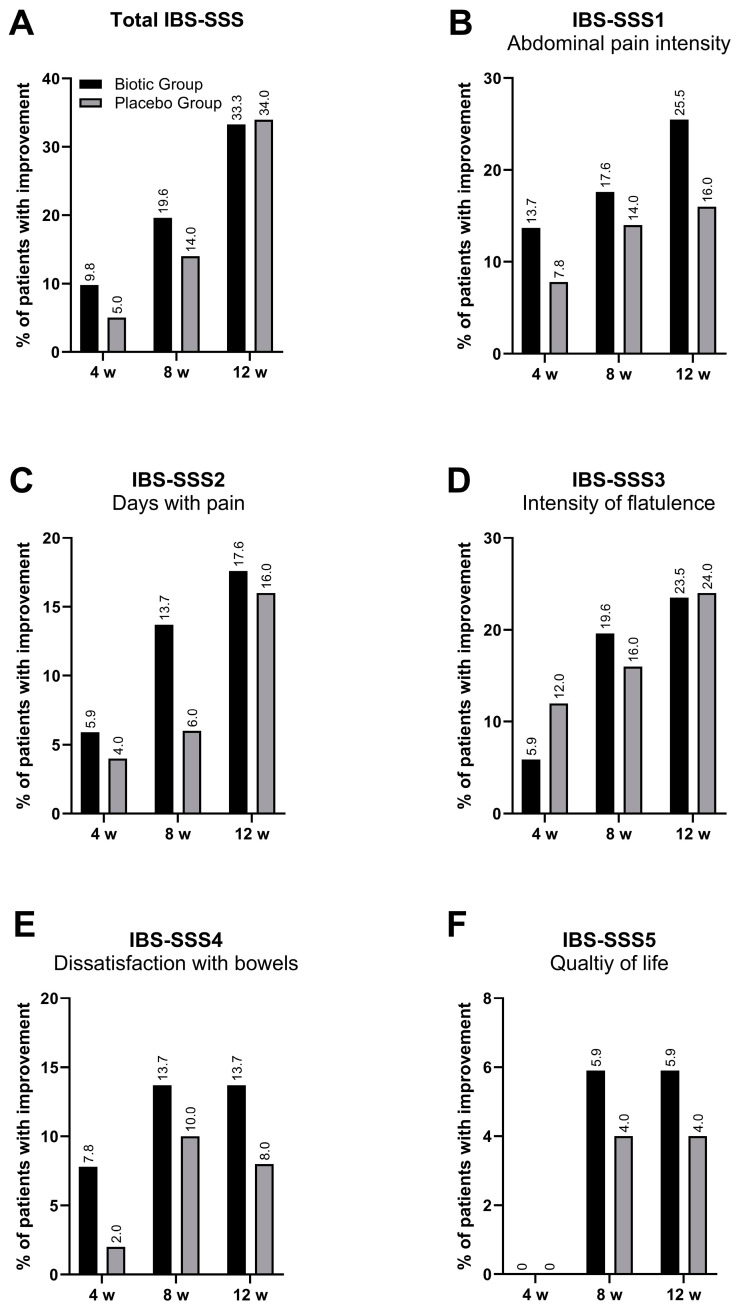
Percentage of patients with an improvement in IBS symptoms as measured by the IBS-SSS at week (w) 4, 8, and 12. Black bars—biotic group, grey bars—placebo group. (**A**)—total IBS-SSS score; (**B**)—abdominal pain intensity (IBS-SSS1 score); (**C**) days with pain (IBS-SSS2 score); (**D**)—intensity of flatulence (IBS-SSS3 score); (**E**)—dissatisfaction with bowels (IBS-SSS4 score); (**F**)—quality of life (IBS-SSS4 score).

Total and specific IBS-SSS subscale scores significantly decreased over time when compared with baseline in both the biotic and placebo group (Supplementary Table S1). Despite a higher proportion of patients reporting improvements (a decrease in score by at least 50% compared with baseline) in both total IBS-SSS or the various IBS-SSS subscales, at weeks 4, 8, or 12 in the biotic group, there were no significant differences between groups (Figure 2).

Similarly, when the effect of the intervention on global improvements was assessed using the IBS-GIS, there were no significant differences between the groups in the percentage of patients reporting improvements (5–7 points on the IBS-GIS).

**Figure 3:**
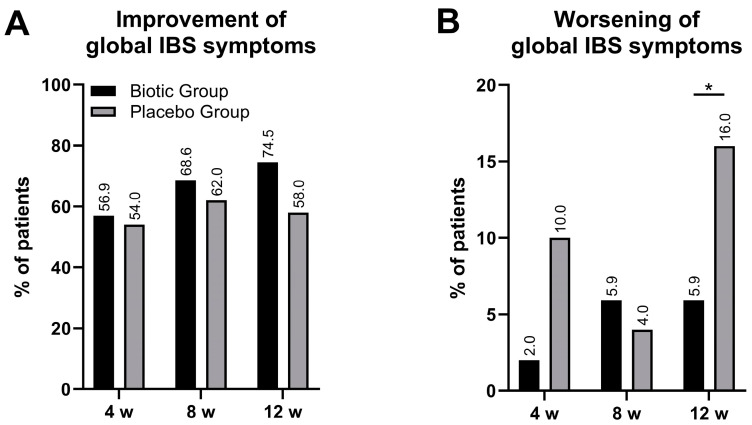
The effect of intervention on global IBS symptoms as measured by the IBS-GIS. The results are presented as (**A**)—percentage of patients reporting an improvement of symptoms (assessed as 5–7 points) and (**B**)—percentage of patients reporting worsening of symptoms (assessed as 1–3 points). Black bars—biotic group, grey bars—placebo group; * *p* < 0.05.

However, the proportion of patients in the biotic group reporting improvement was generally higher (56.9%, 68.6%, and 74.5% at weeks 4, 8, and 12, respectively) than in the placebo group, where it remained at a similar level of 56–58% throughout the intervention (Figure 3A).

A significant difference between groups was observed in the percentage of patients reporting a worsening of global IBS symptoms (1–3 points on the IBS-GIS). In the biotic group, only one patient (1.99%) indicated a worsening of IBS symptoms at 4 weeks, and this number increased to three patients (5.9%) at weeks 8 and 12, while in the placebo group, clinical deterioration was reported by five patients (10.0%) at week 4 and eight patients (16.0%) at week 12, which was significantly higher compared to the biotic group (*p* = 0.015, OR 0.17, CI [0.041–0.71], Figure 3B).

Significant differences vs. baseline in IBS-QOL were observed in the biotic group at weeks 4 and 12, while in the placebo group this was the case only at week 12 (Table 4). No significant differences were observed between the study groups.

### 3.3. Secondary Outcomes

**Figure 4:**
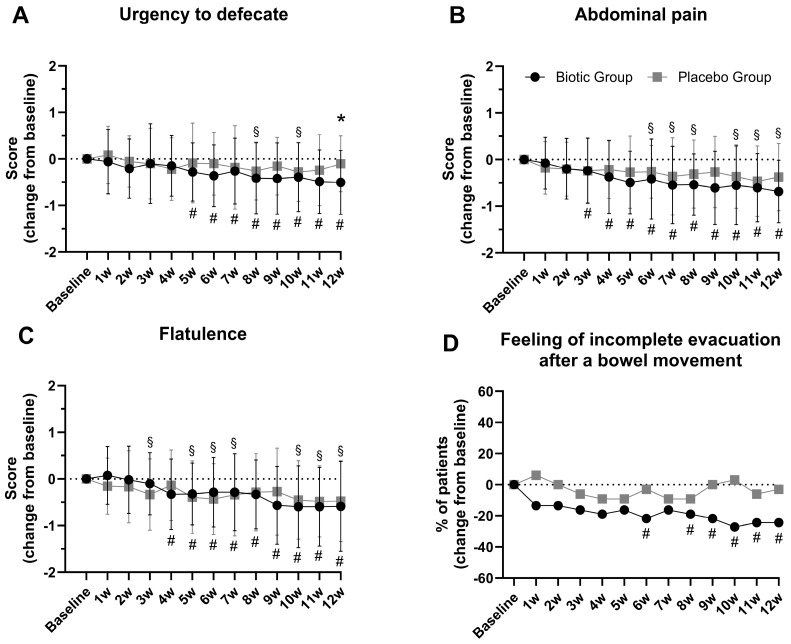
The effect of intervention on patient-recorded symptom scales (5-point Likert). Baseline = one week observation before intervention. Results are presented as change from baseline in means ± SD scores (**A**–**C**) or change from baseline in % of patients who reported ‘feeling of incomplete evacuation after bowel movement’ (**D**) in each week. * *p* < 0.05 between the study groups; # *p* < 0.05 within the biotic group when compared with baseline; § *p* < 0.05 within the placebo group when compared with baseline.

Clinical symptoms were also assessed using patient-recorded symptom scales (Figure 4A–D). While feelings of ‘urgency to defecate’ decreased over time when compared to baseline in both groups, this was more pronounced in the biotic group, with significant differences between groups at week 12 (*p* = 0.015, Figure 4A). Similarly, the decrease in feelings of ‘abdominal pain’ were more pronounced and earlier in onset in the biotic group when compared to baseline; however, there were no differences between groups (Figure 4B). While there was no difference between groups in feelings of flatulence (Figure 4C), there was a difference in the percentage of patients reporting ‘feelings of incomplete evacuation after a bowel movement’ which was significantly reduced when compared to baseline only in the biotic group but not in the placebo group (Figure 4D).

Daily assessment of the number of bowel movements and consistency of stools measured by the patient-recorded BSF scale did not show significant differences between the study groups. There were also no significant differences between the groups in terms of anthropometric parameters and BMI (Supplementary Table S2) or proinflammatory cytokines (Supplementary Table S3).

### 3.4. Safety and Tolerance

There were no significant differences between study groups both in AE incidence as well as AE absolute prevalence (Table 5). Patients reported the following AEs: headache, dizziness, nausea, heartburn, and abdominal distension. No AEs led to the discontinuation of the biotic intervention.

## 4. Discussion

Given the substantial evidence linking microbiome composition to a wide range of diseases and pathologies [31,32], pre-, pro-, and synbiotics are increasingly being used in the treatment of IBS. It is emphasized, however, that further clinical studies are required to strengthen the scientific evidence supporting their clinical efficacy [17]. The current study is the first to evaluate the effect of a combination of a multispecies probiotic, small amounts of prebiotic scFOSs, and microencapsulated butyrate to improve clinical symptoms in patients with IBS. We found that IBS patients supplemented with the biotic preparation over 12 weeks reported statistically significant adequate relief (evaluated using the IBS-AR scale) as early as 4 weeks after the start of the intervention (64.7% vs. 42.0% of patients in the placebo group) and significantly reduced ‘worsening of symptoms’ (evaluated using the IBS-GIS) after 12 weeks of the intervention (5.9% vs. 16.0% in the placebo group). These findings are somewhat consistent with our previous study, in which we used a synbiotic preparation containing the same multispecies probiotic mixture and scFOSs, albeit at higher doses (1 × 10^10^ CFU probiotics and ~1.9 g of scFOSs per day), but without the inclusion of microencapsulated butyrate [18]. In the previous study, we also observed significantly improved IBS-GIS scores at week 8 and primarily a reduction in the total IBS-SSS and in domain-specific scores related to flatulence at 4 and 8 weeks when compared to the placebo. However, there was no effect on adequate relief using the IBS-AR scale [18]. Therefore, when comparing both studies, it can be concluded that the addition of microencapsulated sodium butyrate resulted in comparable efficacy, even with lower dosages of probiotics and scFOSs, thereby maintaining the effectiveness of the intervention. Of note, the exclusion of high doses of scFOSs allowed for a convenient capsule format, unlike the previous mixture, which required sachets.

The beneficial effect of microencapsulated sodium butyrate in IBS may be attributed to its unique physiological properties [33]. Recent research highlights the importance of SCFAs in maintaining intestinal balance, which can be relevant to the pathogenesis of IBS [10]. Particularly, butyrate plays a key role in supporting gastrointestinal health by preventing dysbiosis, protecting the gut barrier, and modulating the immune system and the gut–brain axis [11]. Indeed, the potential mechanism of butyrate’s action in IBS was recently explored in a combination of in vitro and experimental mice models which demonstrated that interleukin-1 receptor-associated kinase 1 (IRAK1) is positively associated with visceral hypersensitivity, while treatment with sodium butyrate reduced this hypersensitivity by inhibiting IRAK1 expression [34]. The efficacy of microencapsulated sodium butyrate was also studied in humans. In a randomized placebo-controlled trial including 66 IBS patients, Banasiewicz et al. [12] assessed the effect of 300 mg of sodium butyrate supplemented daily for 12 weeks using a set of questionnaires measuring the severity and frequency of selected clinical IBS symptoms and subjective improvement of symptoms. They found that after 4 weeks, there was a significant reduction in pain during defecation in the sodium butyrate group which extended to the improvement of urgency and bowel habit at 12 weeks. The reduction of abdominal pain, flatulence, and disordered defecation was, however, not statistically significant. In recently published prospective multicenter trial including a total of 3000 adult IBS patients who were treated with microencapsulated sodium butyrate at a dosage of 150 mg twice a day for 12 weeks, Lewandowski et al. [35] reported a significant (*p* < 0.001) improvement in severity of abdominal pain, flatulence, stool consistency, urgent pressure for bowel movements, and nausea. However, it is necessary to underline that this study was not a placebo-controlled randomized trial and effects of sodium butyrate were evaluated comparing the baseline and end of the intervention. Unfortunately, the lack of a control group in this study does not allow for a clear conclusion of the effect of sodium butyrate, given the relatively large placebo effect that is known in patients with IBS [36,37].

A strong placebo effect was also observed in both our previous [18] and the current study in a range of measurements including, e.g., the IBS-SSS, IBS-QOL, or patient-recorded symptom scales, as evident from significant within-group changes from baseline (Figure 4, Table 4 and Table S1). Additionally, the percentage of patients reporting an improvement of symptoms using the IBS-AR and IBS-GIS was high in the control group, respectively 56% and 58% after 12 weeks of intervention. Such strong placebo effects are often attributed to the high level of care given to patients enrolled in studies, which includes weekly phone calls with interviewers, frequent doctor visits, and access to doctors by phone. However, we cannot rule out an effect caused by the maltodextrin which was used as the placebo in the current study. Almutairi et al. [38] recently evaluated the effect of maltodextrin on the gut microbiome and host physiology by including 70 randomized controlled trials in a systematic review. Their findings showed that maltodextrin consumption often led to changes in gut microbiota composition in 61.8% of the trials, particularly affecting the *Firmicutes* and *Bacteroidetes* phyla, as well as *Lactobacillus* and *Bifidobacterium* species. Additionally, maltodextrin influenced immunological and inflammatory markers, along with gut function and permeability, in 25.6% of the studies. Changes in microbial metabolites were observed in 19% of the trials. Regarding butyrate, the results were mixed. In one study, overweight individuals who consumed maltodextrin providing 15% of their energy intake for three weeks showed an increase in fecal butyrate levels [39], while in another study with lower dosages over a shorter duration, butyrate levels decreased in healthy individuals [40]. Given that we believe that intestinal dysbiosis plays a key role in the pathogenesis of IBS, the strong placebo effect observed in our study may be attributable to maltodextrin’s direct impact on the gut microbiota. Unfortunately, we did not assess the composition of the gut microbiota or the microbial metabolome, so we cannot determine if, or how, maltodextrin influences the microbiome of IBS patients.

We also analyzed the impact of the biotic preparation on anthropometric measurements and BMI as a secondary outcome given that SCFAs, including butyrate, are known to contribute about 5–10% of the total dietary energy supply in humans and may promote lipogenesis [41]. However, our 12-week observation showed no differences in these parameters between the study groups, suggesting that a daily dose of 300 mg of sodium butyrate does not affect energy homeostasis or body weight.

Another secondary outcome was the effect of the biotic preparation on inflammatory markers including IL-6 and CCL-4 in patients’ sera, as those appear to be an important mediator in IBS patients. For example, Bennet et al. [42] demonstrated that while the global cytokine profiles of IBS patients and healthy controls overlapped, the cytokine levels in IBS patients showed greater variation. These patients exhibited distinct cytokine profiles, including elevated serum levels of IL-6 and IL-8 and reduced levels of IFN-γ. A systematic review and meta-analysis investigating circulating IL-6 levels in IBS found that IL-6 levels are higher in IBS patients compared to controls [43]; however, when categorizing based on IBS subtypes, IL-6 levels were significantly higher only in IBS-D patients compared to healthy controls, but not in patients with IBS-C and IBS-M. In contrast, a recent study with oral administration of *Limosilactobacillus reuteri* ATCC PTA 6475 to IBS-D patients showed a trend toward reduced serum IL-6 levels compared to the placebo group (*p* = 0.052) [44]. In our study, we did not observe any significant differences in IL-6 and CCL-4 between the study groups and there was also no effect of IBS subtypes on cytokine levels, suggesting that the biotic intervention did not affect the serum concentrations of these proinflammatory mediators. However, it is possible that the absence of a reduction in cytokine production following the biotic mixture is related to the fact that IBS progression primarily depends on gut mucosal immunity [45]. Assessing cytokines in stool or biopsy samples may therefore provide more relevant insights than evaluating serum cytokines.

### Strengths and Limitations of the Study

The current study was designed as a state-of-the-art, randomized, double-blind, placebo-controlled clinical study involving adult IBS patients diagnosed on the basis of the Rome IV criteria. Patients were closely monitored through frequent scheduled visits, daily recording of clinical symptoms, and adverse event reporting in patients’ diaries, as well as monitoring of patients by telephone interviewers. Nevertheless, there are a number of limitations that should be noted. First, we did not assess the biotic preparation’s effect on the composition and function of the gut microbiome, which would have provided deeper insight into the mechanisms underlying the observed clinical outcomes. Secondly, we did not account for the relatively strong placebo effect often seen in clinical trials involving IBS patients. Other studies have implemented experimental designs that include a placebo run-in period, excluding participants who exhibit a particularly strong response based on predefined outcome measures. Finally, since IBS is a chronic condition, a longer intervention period with follow-up visits after discontinuing supplementation could have offered valuable insights into the long-term effects of biotics in IBS.

## 5. Conclusions

This randomized, double blind, placebo-controlled clinical study involving a total of 120 adult patients with IBS-D, IBS-C, and IBS-M subtypes shows that supplementing a biotic mixture including microencapsulated sodium butyrate, probiotics, and small amounts of scFOS for 12 weeks is safe, well tolerated, and effective in improving gastrointestinal symptoms in patients with IBS. As early as 4 weeks after commencing the intervention, there was a significantly higher number of patients in the biotic group reporting adequate relief of symptoms (based on IBS-AR) when compared with the placebo group. Moreover, at 12 weeks, fewer patients in the biotic group reported a ‘worsening of symptoms’ (based on IBS-GIS). There was no significant differences between groups in IBS-QOL or IBS-SSS or any of the secondary outcome measures except patient-recorded ‘urgency to defecate’ at week 12, which was significantly lower in the biotic group. These data collectively indicate that the capsule-based biotic mixture may be beneficial in managing gastrointestinal symptoms in patients with IBS.

## 6. Future Perspective

To gain a deeper mechanistic understanding of biotic mixtures including pre- and probiotics as well as microencapsulated butyrate in IBS patients, further research is needed to explore their impact on gut microbiome composition, metabolites, and mucosal immune activation. Additionally, comparing the effects of the biotic mixtures across different IBS subtypes could provide valuable insights into their efficacy in treating functional gastrointestinal disorders.

## Figures and Tables

**Table 1:** Specification and doses of the biotic product administered to IBS patients.

Active Ingredient	Dose per Capsule *
Microencapsulated sodium butyrate	300 mg (equal to 150 mg sodium butyrate)
Probiotic strains	
*Bifidobacterium lactis* DSM 32269	5.2 × 10^8^ CFU
*Bifidobacterium longum* DSM 32946	1.0 × 10^8^ CFU
*Bifidobacterium bifidum* DSM 32403	1.0 × 10^8^ CFU
*Lactobacillus acidophilus* DSM 32418	1.4 × 10^8^ CFU
*Lacticaseibacillus rhamnosus* DSM 26357	1.4 × 10^8^ CFU
Total	1 × 10^9^ CFU
Prebiotic	
scFOSs	64 mg

* Patients were instructed to take two capsules daily, consumed orally 30 min after meals—one capsule after breakfast and one after dinner—for a duration of 12 weeks. CFU, colony forming units; scFOSs, short chain fructooligosaccharides.

**Table 2:** Patients’ characteristics.

	Biotic Group (n = 51) n (%) or Mean ± SD	Placebo Group (n = 50) n (%) or Mean ± SD	*p*-Value
Gender			
Female	40 (78.4%)	35 (70.0%)	0.334
Male	11 (21.6%)	15 (30.0%)	0.334
Age in years	40.0 ± 15.0	43.7 ± 14.5	0.123
BMI	24.4 ± 4.5	24.3 ± 3.6	0.956
IBS type			
IBS-D	19 (37.3%)	24 (48.0%)	0.313
IBS-C	15 (29.4%)	14 (28.0%)	0.383
IBS-M	17 (33.3%)	12 (24.0%)	0.078
IBS severity *			
Moderate	30 (58.8%)	32 (64.0%)	0.593
Severe	21 (41.2%)	18 (36.0%)	0.593
Total IBS-SSS score	272.0 ± 74.3	272.9 ± 64.5	0.796

* IBS severity was assessed with the use of IBS-Severity Scoring System (IBS-SSS); severe IBS when IBS-SSS was >300 points and moderate when IBS-SSS was >175 and ≤300; BMI, body mass index; IBS-D, diarrhea-predominant IBS; IBS-C, constipation-predominant IBS; IBS-M, mixed type of bowel habits.

**Table 3:** The effect of intervention on adequate relief in IBS patients assessed using the IBS-AR scale.

Time of Intervention in Weeks (w)	Adequate Relief n (%)		Odds Ratio [95% CI]	*p*-Value
	Biotic Group (n = 51)	Placebo Group (n = 50)		
4 w	33 (64.7%)	21 (42.0%)	2.53 [1.13–5.65]	0.023
8 w	38 (74.5%)	31 (62.0%)	1.79 [0.77–4.19]	0.179
12 w	37 (72.5%)	28 (56%)	2.07 [0.90–7.77]	0.085

The results present the number of IBS patients reporting over the past week adequate relief of IBS symptoms. CI, confidence interval.

**Table 4:** The effect of intervention on quality of life assessed with the IBS-QOL.

Groups	Baseline	At Week 4 of Intervention				At Week 12 of Intervention			
	Mean ± SD	Mean ± SD	Change from Baseline	*p*-Value Within Group *	*p*-Value vs. Placebo	Mean ± SD	Change from Baseline	*p*-Value Within-Group *	*p*-Value vs. Placebo
Biotic group	50.4 ± 20.4	44.0 ± 20.4	6.4 ± 9.5	<0.00001	0.830	39.6 ± 20.4	10.7 ± 14.9	<0.00001	0.730
Placebo Group	48.4 ± 18.2	44.9 ± 20.9	3.5 ± 16.6	0.065	N.A.	41.0 ± 20.8	7.4 ± 18.5	0.002	N.A.

* *p*-value within group in comparison with baseline; N.A., not available.

**Table 5:** Incidence and absolute prevalence of adverse events reported by patients.

Adverse Events	Incidence (%)				Prevalence (%)			
	Biotic Group	Placebo Group	*p*-Value	Odds Ratio [95% CI]	Biotic Group	Placebo Group	*p*-Value	Odds Ratio [95% CI]
Headache	9.8	6.0	0.483	1.7	4.2	5.4	0.732	0.79
				[0.38–7.54]				[0.20–3.05]
Dizziness	5.9	8.0	0.676	0.72	3.7	5.0	0.70	0.74
				[0.15–3.39]				[0.16–3.41]
Nausea	11.8	6.0	0.318	2.1	11.9	9.5	0.618	1.28
				[0.49–8.86]				[0.48–3.43]
Heartburn	2.0	0	0.668	3.0	1.2	0	0.498	3.01
				[0.12–75.41]				[0.12–75.60]
Abdominal distension	3.9	6.0	0.633	0.64	3.6	2.4	0.652	1.51
				[0.10–4.0]				[0.25–9.33]

CI, confidential interval.

## Data Availability

The study protocol is available at https://clinicaltrials.gov/study/NCT05013060 (accessed on 21 October 2024) and has been published [19]. The data are available on request from the corresponding author. The data are not publicly available due to privacy protection.

## Supplementary Materials

The following supporting information can be downloaded at: https://www.mdpi.com/article/10.3390/jcm14010006/s1, Table S1. The effect of biotic intervention on changes in IBS-SSS score; Table S2. The effect of intervention on anthropometric measurements; Table S3. The effect of intervention on cytokine levels in patients’ sera.
