# Supplementary material for: Efficacy and Safety of a Mixture of Microencapsulated Sodium Butyrate, Probiotics, and Short Chain Fructooligosaccharides in Patients with Irritable Bowel Syndrome—A Randomized, Double-Blind, Placebo-Controlled Study
Source: J Clin Med. 2024 Dec 24;14(1):6. doi: 10.3390/jcm14010006 (PMC11720862; doi:10.3390/jcm14010006)
Supplement: Supplementary file 1 [file jcm-14-00006-s001.zip › jcm-3339650-supplementary.pdf]

Table S1. The effect of biotic intervention on changes in IBS-SSS score.

Groups	Baseline	At week 4 of intervention		At week 8 of intervention		At week 12 of intervention	
	Mean±SD	Change from baseline	*p*-value within-group	Change from baseline	*p*-value within-group	Change from baseline	*p*-value within group±
**Total IBS-SSS**							
Biotic	272.7±73.8	-44.3±77.9	0.0002	-81.3±99.0	<0.00001	-86.3±110.6	<0.00001
Placebo	272.9±64.5	-51.6±50.4	<0.00001	-77.1±56.5	0.0001	-86.7±83.7	0.0002
**IBS-SSS 1 (the severity of pain)**							
Biotic	48.0±22.3	-9.8±21.8	0.0027	-17.2±25.1	<0.00001	-19.1±26.6	<0.00001
Placebo	51.1±20.6	-13.6±19.0	<0.00001	-20.1±20.6	<0.00001	-21.6±21.2	<0.00001
**IBS-SSS 2 (the frequency of pain)**							
Biotic	33.1±23.2	-4.2±23.6	0.1516	-9.3±29.6	0.0091	-9.0±30.2	0.0322
Placebo	34.0±21.3	-6.5±16.6	0.0135	-10.5±18.3	0.0002	-12.5±24.4	0.0008
**IBS-SSS 3 (the severity of flatulence)**							
Biotic	54.2±22.8	-7.2±22.3	0.0227	-17.3±27.6	<0.00001	-18.9±30.0	<0.00001
Placebo	44.0±27.0	-10.0±20.2	0.0017	-11.5±22.7	0.0018	-12.0±30.0	0.0071
**IBS-SSS 4 (dissatisfaction with bowel habit)**							
Biotic	71.5±15.8	-12.9±22.9	0.0003	-20.7±24.0	<0.00001	-20.6±27.2	<0.00001
Placebo	77.6±18.9	-15.5±18.2	<0.00001	-23.1±23.6	0.0001	-24.7±22.2	0.0002
**IBS-SSS 5 (quality of life)**							
Biotic	65.8±15.8	-10.3±16.6	0.0001	-10.3±16.6	<0.00001	-18.5±22.1	<0.00001
Placebo	66.2±19.1	-6.0±19.0	0.0531	-11.9±21.0	0.0003	-15.8±23.4	0.0001

The severity of IBS symptoms was assessed using the IBS-SSS survey before treatment (baseline), and at week 4, 8 and 12 after intervention. A score reduction was related to symptom amelioration. The results are presented as means ± standard deviation (SD) of changes in IBS-SSS scores from baseline. $p<0.05$ presents significant differences within groups. There was no significant differences between groups.

Table S2. The effect of intervention on anthropometric measurements.

**Parameters**	**Biotic group**		***p*-value within biotic group**	**Placebo group**		***p*-value within placebo group**	***p*-value between groups**	
	Before intervention (Baseline)	At week 12 of intervention		Before intervention (Baseline)	At week 12 of intervention		Before intervention (Baseline)	At week 12 of intervention
**Weight (kg)**	68.4±14.8	68.8±14.2	0.2907	70.0±12.5	70.4±13.0	0.3222	0.5508	0.5508
**Height (m)**	1.67±0.1	1.67±0.1	0.3569	1.70±0.1	1.70±0.1	0.8184	0.1779	0.1617
**Body Mass Index (BMI)**	24.4±4.5	24.8±4.6	0.1843	24.3±3.6	24.3±3.8	0.9255	0.8685	0.5641
**Arm circumference (cm)**	27.6±4.5	27.9±4.3	0.1034	27.8±4.3	27.7±4.1	0.2054	0.8279	0.8236
**Calf circumference (cm)**	35.8±4.2	36.1±3.6	0.3528	36.2±3.9	35.8±3.2	0.1581	0.6609	0.6424
**Waist circumference (cm)**	82.6±13.4	82.9±13.	0.4534	82.90±11.3	82.8±11.5	0.2191	0.8997	0.7874
**Hip circumference (cm)**	99.3±9.0	99.2±9.6	0.9058	100.7±9.9	99.7±9.6	0.0723	0.4631	0.7983
**Waist to hip ratio**	0.83±0.09	0.83±0.09	0.3416	0.82±0.08	0.82±0.08	0.9261	0.7032	0.5613

The results are presented as mean±standard deviation.

Table S3. The effect of intervention on cytokine levels in patients'sera.

**Cytokine**	**Biotic group**		**p-value within biotic group**	**Placebo group**		**p-value within placebo group**	***p*-value between groups**	
	Before intervention (Baseline)	At week 12 of intervention		Before intervention (Baseline)	At week 12 of intervention		Before intervention (Baseline)	At week 12 of intervention
**IL-6 (pg/ml)**	5.7±4.4	5.3±2.7	0.7480	6.5±5.7	6.4±3.3	0.8623	0.2394	0.0971
**CCL-4 (pg/ml)**	236.1±279.2	251.7±315.7	0.6170	312.1±418.3	309.4±417.9	0.7868	0.3305	0.4728

The results are presented as mean±standard deviation.
